# Differential Cytokine-Induced Responses of Polarized Microglia

**DOI:** 10.3390/brainsci11111482

**Published:** 2021-11-10

**Authors:** Priyanka Chauhan, Wen S. Sheng, Shuxian Hu, Sujata Prasad, James R. Lokensgard

**Affiliations:** 1 Neurovirology Laboratory, Department of Medicine, University of Minnesota Medical School, Minneapolis, MN 55455, USA; guptap@umn.edu (P.C.); sheng008@umn.edu (W.S.S.); huxxx031@umn.edu (S.H.); prasads@umn.edu (S.P.); 2 3-107 Microbiology Research Facility, University of Minnesota, 689 23rd Avenue S.E., Minneapolis, MN 55455, USA

**Keywords:** microglia, cytokines, microglial polarization, programmed death-ligand 1 (PD-L1)

## Abstract

The role of select pro- and anti-inflammatory mediators in driving microglial cell polarization into classically (M1), or alternatively, (M2) activated states, as well as the subsequent differential responses of these induced phenotypes, was examined. Expression of PD-L1, MHC-II, MHC-I, arginase 1 (Arg-1), and inducible nitric oxide synthase (iNOS) was assessed using multi-color flow cytometry. We observed that both pro- and anti-inflammatory mediators induced PD-L1 expression on non-polarized microglia. Moreover, IFN-γ stimulated significant MHC class I and II expression on these cells. Interestingly, we observed that only IL-4 treatment induced Arg-1 expression, indicating M2 polarization. These M2 cells were refractory to subsequent depolarization and maintained their alternatively activated state. Furthermore, PD-L1 expression was significantly induced on these M2-polarized microglia after treatment with pro-inflammatory mediators, but not anti-inflammatory cytokines. In addition, we observed that only LPS induced iNOS expression in microglial cells, indicating M1 polarization. Furthermore, IFN-γ significantly increased the percentage of M1-polarized microglia expressing iNOS. Surprisingly, when these M1-polarized microglia were treated with either IL-6 or other anti-inflammatory cytokines, they returned to their non-polarized state, as demonstrated by significantly reduced expression of iNOS. Taken together, these results demonstrate differential responses of microglial cells to mediators present in dissimilar microenvironments.

## 1. Introduction

It is well-established that upon infection or insult within the brain there is a secretion of a wide array of cytokines and chemokines by both immune and non-immune cells [1]. These low molecular weight polypeptides invigorate the cells and draw them towards sites of neuroinflammation. They can act either at the site they emanated (autocrine effect) or on neighboring cells (paracrine fashion), or even on remote cells (endocrine effect/systemic effect) [2]. It is also common that individual cytokines have varying effects on different cell types (pleiotropy). Cytokines are redundant in their function and are often produced in a cascade (i.e., one triggers its target cells to make additional cytokines, which can work synergistically or antagonistically). They modulate cell-to-cell interactions that are directly involved in the advancement of disease pathogenesis [3,4]. Various cytokines are secreted within the brain, which can be either pro-inflammatory or anti-inflammatory [2]. Pro-inflammatory cytokines are secreted by activated cells and are involved in upregulating inflammatory responses. There is evidence suggesting that certain pro-inflammatory cytokines are involved in neuroinflammation-induced sensitization of the brain, such as interleukin (IL)-1β, IL-6, and tumor necrosis factor (TNF)-α [5,6]. IL-1β is primarily produced by macrophages as well as non-immune cells, such as fibroblasts and endothelial cells, during cell injury or inflammation [2,7]. IL-6 is known to be involved in microglial activation and plays a central role in the development of neuropathic pain following a peripheral nerve injury [8]. TNF-α, is another pro-inflammatory cytokine that plays important roles in both inflammatory and neuropathic hyperalgesia [2,9]. Receptors for TNF-α are present on both neurons and glial cells. Interferon (IFN)-γ is mainly produced by natural killer cells or innate lymphoid type 1 cells [10,11]. It stimulates anti-viral immunity through its regulatory effects on the innate immune response and serves as a link between innate immune responses and the activation of adaptive immunity. It induces major histocompatibility complex (MHC)-I and MHC-II expression on T-cells and plays an important role in macrophage activation [12]. In contrast, anti-inflammatory cytokines are immune-regulatory molecules that regulate pro-inflammatory cytokine responses [2,13]. Major anti-inflammatory cytokines include IL-1 receptor antagonist, IL-4, and IL-10. Transforming growth factor (TGF)-β is categorized as either anti-inflammatory or pro-inflammatory cytokines under various circumstances [2].

MHC class I and class II molecules are upregulated on target cells to present peptides to the immune system for their effective clearance [14]. MHC-I is expressed on all nucleated cells and presents peptides derived from intracellular proteins that can be either the cell’s own endogenous proteins or from exogenous sources (i.e., viral peptides) [15]. This is essential to maintain brain homeostasis, but if uncontrolled, it can result in neuroinflammation and bystander damage to the organ. Hence, there is an engagement of programmed death (PD)-1 (on T-lymphocytes) with PD-L1 (B7-H1; CD274) (on glial cells) for functional suppression of activated T-cells [16]. PD-1/PD-L1 is a well-known negative immune checkpoint pathway that modulates T-cell effector function by decreasing their proliferative capacity, cytokine production, and cytotoxic effector functions, and thereby, control detrimental bystander damage [17].

Microglial cells are brain-resident cells that regulate brain development, maintenance, and injury repair [18,19]. As specialized scavengers, they are responsible for the elimination of foreign antigens that may endanger the central nervous system (CNS) [20]. Moreover, as the primary source of pro-inflammatory cytokines, microglia can induce a broad spectrum of immune responses [21]. Microglial activation has been long associated with neuroinflammation and alterations in microglia functionality can have implications in brain development and neurodegeneration [18,22,23]. Microglial activation is often classified as either classical (M1) or alternative (M2), following the paradigm used for macrophages [24,25]. M1 polarization is a pro-inflammatory state typically invigorated by the simultaneous triggering of toll-like receptors (TLR) and IFN-γ signaling pathways. M1-polarized microglia express nicotinamide adenine dinucleotide phosphate (NADPH) oxidase, which generates superoxide and reactive oxygen species (ROS), as well as inducible nitric oxide synthase (iNOS), which converts L-arginine into nitric oxide (NO) for the defense of the brain [19]. Hence, iNOS expression by microglial cells indicates an M1-polarized state. On the other hand, M2 activation describes the anti-inflammatory and repair activities of microglia [26]. M2-polarized microglial cells promote the release of anti-inflammatory cytokines, such as IL-10 and TGF-β, and produce arginase 1 (Arg-1), which promotes the conversion of L-arginine into L-ornithine and polyamines for brain repair [26,27].

In our previous studies, we have shown that PD-L1 is upregulated on microglial cells following viral brain infection and, correspondingly, PD-1 is expressed on brain-infiltrating CD8+ T-cells [28,29]. The expression of PD-L1 on microglia is thought to govern the activity of these brain-infiltrating anti-viral T-cells to possibly limit damaging encephalitic responses [30]. In this study, we investigated the role of select, prototypical cytokines in polarizing primary murine microglial cells to classically (M1) or alternatively activated (M2) states; and examined the subsequent differential responses of these induced phenotypes (by studying the upregulation of PD-L1 and MHC molecules). The cytokines selected were IFN-γ, TNF-α, IL-1β, and IL-6 as mediators of pro-inflammation, and IL-4, IL-10, and TGF-β as anti-inflammatory stimulants. LPS was used as a positive control.

## 2. Materials and Methods

### 2.1. Ethical Statement

This study was carried out according to guidelines for the Care and Use of Laboratory Animals of the National Institutes of Health. The protocol was approved by the University of Minnesota’s Institutional Animal Care and Use Committee (Protocol Number: 2001-37741A). All animals were routinely cared for according to Research Animal Resources (RAR) guidelines. Animals were euthanized after isoflurane inhalation, whenever required and efforts were made to ameliorate suffering.

### 2.2. Experimental Animals

C57BL/6 (7-10 d old) mice were used for the isolation of microglial cells. Pathogen-free C57BL/6 (JAX stock#000664) mice were purchased from The Jackson Laboratory (Bar Harbor, Maine). Animals were lodged in individually ventilated cages and were provided with food and water ad libitum at RAR, University of Minnesota.

### 2.3. Reagents

Dulbecco’s Modified Eagle Medium (DMEM), fetal bovine serum (FBS), penicillin, streptomycin, LPS, and trypsin were purchased from Sigma-Aldrich (St. Louis, MO, USA). Gentamicin, Fungizone^®^, and IL-4 were bought from Thermo Fisher Scientific (Waltham, MA, USA). IFN-γ, TNF-α, IL-1β, IL-6, IL-10, and TGF-β were bought from R&D Systems (Minneapolis, MN, USA). Antibodies used for flow cytometry: CD45-APCeFluor780, CD11b-AF700, MHC-I-APC, PD-L1-PE-Cy7, Arg-1-eFluor450, and iNOS-PE were procured from Thermo Fisher Scientific while MHC-II-BV510 was purchased from Biolegend (San Diego, CA, USA). Cytofix/Cytoperm kit used for cell permeabilization was also purchased from Thermo Fisher Scientific.

### 2.4. Primary Murine Microglial Cell Cultures

Murine microglial cells from 7 – 10 d old mice were isolated using the Adult Brain Dissociation Kit followed by CD11b microbeads (Miltenyi Biotec, Gaithersburg, MD, USA). Briefly, brains were dissected, weighed, and enzymatically digested using the dissociation kit for 30 min at 37 °C (>100 mg tissue). Further tissue processing was carried out at 4 °C. The cell suspension was passed through a 70 μm cell strainer and the supernatant was centrifuged at 300× *g* for 10 min. Cells were resuspended in cold D-PBS and tissue debris was removed by using Debris Removal Solution and overlayed with an appropriate amount of cold D-PBS. Cells were centrifuged at 3000× *g* for 10 min and the top two phases (containing debris) were discarded. Cells were washed and further processed for removing red blood cells (RBCs). For this, the cell pellet was resuspended in cold 1X RBC Removal Solution and refrigerated for 10 min. Cells were then washed and resuspended in an appropriate volume of cold magnetic buffer (PBS + 0.5% BSA). To isolate the microglia, cells were incubated with 10 µL of CD11b microbeads per 10^7^ cells/90 µL for 15 min in the refrigerator. CD11b^+^ cells were separated in a magnetic field using MS columns. The amounts of antibodies and the buffers were calculated based on the number of cells obtained after RBCs removal, using the manufacturer’s guidelines. Purified microglial cells stained >95% positive for the microglial phenotype (CD45^int^CD11b^+^).

### 2.5. In-Vitro Stimulation and Intracellular Staining

Microglial cells were counted and 0.5 × 10^6^ cells were plated in 96-well suspension plates in DMEM containing 10% FBS, penicillin (100 U/mL), streptomycin (100 μg/mL), gentamicin (50 μg/mL) and Fungizone^®^ (250 pg/mL) and incubated at 37 °C overnight. Cells were then stimulated with select pro- and anti-inflammatory mediators (LPS, 10 ng/mL; IFN-γ, 10 ng/mL; TNFα, 20 ng/mL; IL-1β, 10 ng/mL; IL-4, 100 ng/mL; IL-10, 30 ng/mL, TGF-β, 10 ng/mL, and IL-6, 100 ng/mL). Then cells were trypsinized and then surface stained for CD45-APCeFluor780, CD11b-AF700, MHC-II-BV510, MHC-I-APC, and PD-L1-PE-Cy7 prior to permeabilization and fixation using Cytofix/Cytoperm kit. Cells were then stained for Arg-1-eFluor450 and iNOS-PE, according to the manufacturer’s protocol.

### 2.6. In-Vitro Stimulation of M1 and M2-Activated Microglia

Microglial cells were treated with LPS or IL-4 to polarize them to M1- or M2-activated states for 48 h. The polarized cells were then treated with select pro- and anti-inflammatory cytokines for 24 h, as discussed previously. Cells were then trypsinized and collected for flow cytometry.

### 2.7. Real-Time RT-PCR

Total RNA (0.5–1.0 μg) from primary murine microglial cells cultured in 12-well tissue culture plates was extracted using an RNeasy Lipid Tissue Mini Kit (Qiagen, Valencia, CA, USA) according to the manufacturer’s instructions. After DNase treatment, cDNA was synthesized from total RNA using Superscript III reverse transcriptase (Invitrogen, now Thermo Fisher Scientific, Waltham, MA, USA) and oligo d(T)_12-18_ primers (Gene Link, Elmsford, NY, USA). Real-time RT-PCR was performed with the TB Green^®^ Advantage^®^ qPCR Premix (Takara Bio, San Jose, CA, USA). The qPCR conditions were: 1 denaturation cycle at 95 °C for 10 s; 40 amplification cycles of 95 °C for 10 s, 60 °C annealing for 10 s, and elongation at 72 °C for 10 s; followed by 1 dissociation cycle (Bio-Rad CFX96 Real-Time System, Bio-Rad, Hercules, CA, USA). The following primers were used: sense 5′-GCAACCTGTGTCCTTTCTCCTGAA-3′ and antisense 5′-GGTCTCTTCCATCACCTTGCCAAT-3′ for Arg-1; sense 5′-TGGCCACCTTGTTCAGCTACG-3′ and antisense 5′-GCCAAGGCCAAACACAGCATAC-3′ for iNOS; and sense 5′-TGCTCGAGATGTCATGAAGG-3′ and antisense 5′-AATCCAGCAGGTCAGCAAAG-3′ for hypoxanthine guanine phosphoribosyl transferase (HPRT), as a housekeeping gene. The relative expression levels were normalized to HPRT using the 2^−∆∆Ct^ method [31].

### 2.8. Statistical Analyses

GraphPad Prism software was used to determine statistical significance (version 9.2; Graphpad Software, La Jolla, CA, USA). For comparing groups, Dunnett’s multiple comparisons test and a two-tailed unpaired T-test were employed. A *p*-value < 0.05 was regarded as significant.

## 3. Results

### 3.1. PD-L1 Expression on Non-Polarized Microglial Cells

**Figure 1:**
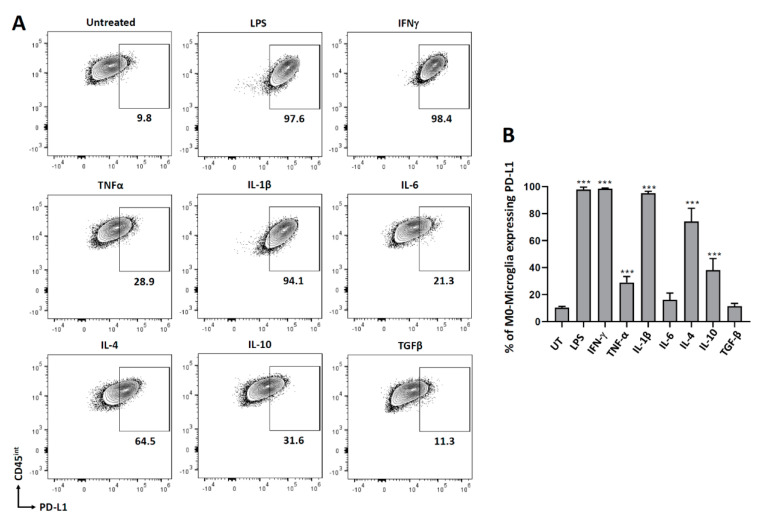
PD-L1 expression on non-polarized (M0) microglial cells. Microglia were isolated from 7-10 d old pups using the Miltenyi mouse adult dissociation kit followed by CD11b isolation. Cells were treated with various pro- and anti-inflammatory mediators for 24 h and then trypsinized to stain for PD-L1. (**A**) Representative contour plots showing microglial cells (CD45^int^) expressing PD-L1. (**B**) Graphical representation of microglial cells expressing PD-L1 after 24 h of treatment. UT-untreated control. Experiments were carried out three times with duplicate wells and presented as mean ± SE. *** *p* < 0.001.

To investigate the role of various pro- and anti-inflammatory cytokines in modulating the expression of PD-L1 on microglial cells, we isolated microglia from C57BL/6 mice (d7-10). Microglial cells were rested overnight in the incubator followed by the addition of various cytokines (IFN-γ, TNF-α, IL-1β, IL-6, IL-4, IL-10, and TGF-β), as well as LPS as a control. After 24 h of treatment, microglial cells were trypsinized, stained for PD-L1, and then analyzed by flow cytometry. We observed that both pro- and anti-inflammatory mediators induced PD-L1 expression on microglial cells, except IL-6 and TGF-β (Figure 1). More than 95% of microglial cells expressed PD-L1 on treatment with LPS (97.8 ± 0.87%), IFN-γ (98.5 ± 0.2%), and IL-1β (95.1 ± 1.0%). 28.8 ± 2.3% of microglial cells expressed PD-L1 after treatment with TNF-α. When the cells were treated with IL-4 and IL-10, 74.1 ± 4.9% and 38.2 ± 4.2% expressed PD-L1 on their surface, respectively.

### 3.2. Expression of MHC Molecules on Non-Polarized Microglial Cells

**Figure 2:**
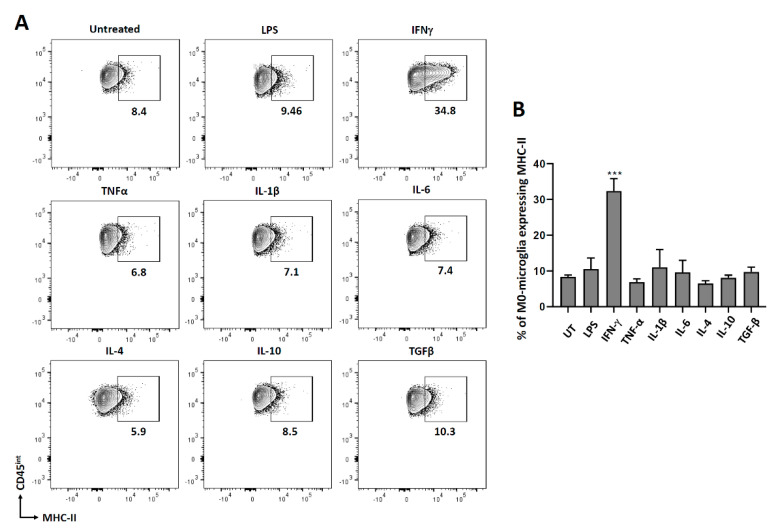
MHC-II expression on non-polarized (M0) microglial cells. Microglial cells were isolated as described. The cells were then treated with several pro- and anti-inflammatory cytokines for 24 h. They were trypsinized and then immuno-stained for MHC-II for flow cytometry. (**A**) Representative contour plots showing microglia (CD45^int^) expressing MHC-II. (**B**) Graphical representation of MHC-II expression after 24 h of treatment. UT-untreated control. Experiments were carried out three times with duplicate wells. *** *p* < 0.001.

**Figure 3:**
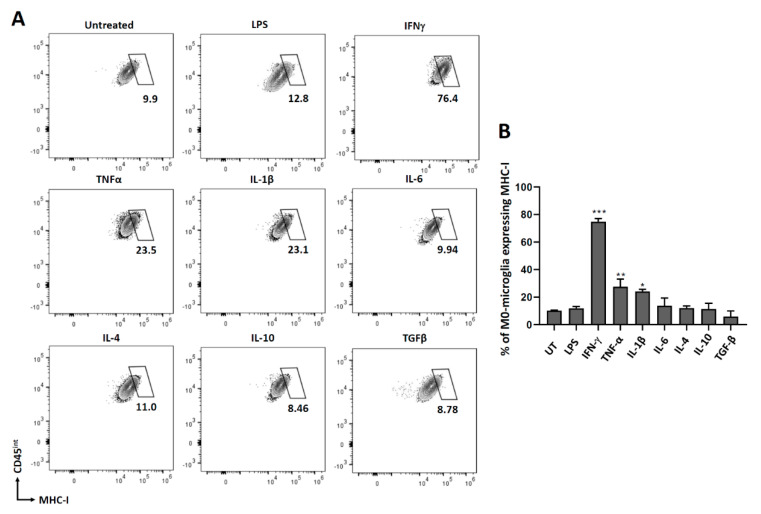
MHC-I expression on non-polarized (M0) microglial cells. Microglia were treated with pro- and anti-inflammatory mediators for 72 h, trypsinized, and stained for MHC-I. (**A**) Representative contour plots showing microglial cells (CD45^int^) expressing MHC-I. (**B**) Graphical representation of MHC-I expression after 72 h of treatment. UT-untreated control. Experiments were carried out three times with duplicate wells. * *p* < 0.05; ** *p* < 0.01; *** *p* < 0.001.

We further went on to investigate the role of different cytokines in modulating microglial activation by analyzing MHC molecules. Both MHC class I and class II are upregulated on antigen-presenting cells to present the peptides to the immune cells for their effective clearance. For this experiment, microglial cells were treated with different cytokines as described previously, and 24 h later, cells were stained for both MHC class I and class II molecules. We observed that only IFN-γ induced significant MHC-II expression on microglia after 24 h of treatment (Figure 2). Since we did not observe any MHC class I expression at 24 h or 48 h post-IFN-γ treatment (Supplementary Figure S1), we analyzed its expression at 72 h post-treatment. We observed that IFN-γ, TNF-α, and IL-1β all induced significant MHC-I expression on microglial cells after 72 h of treatment compared to the untreated control (Figure 3).

### 3.3. Polarization of Microglial Cells to Alternatively Activated State (M2)

**Figure 4:**
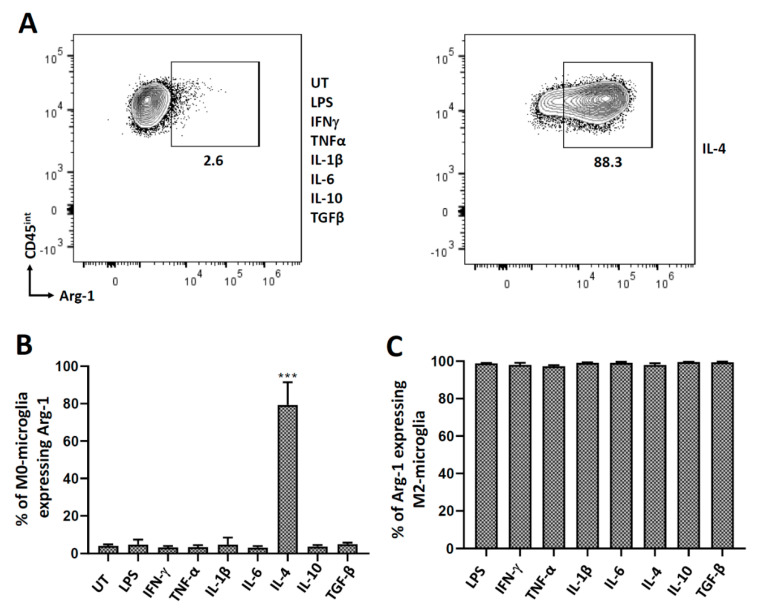
Arginase-1 (Arg-1) expression in microglial cells. Non-polarized, primary murine microglia were treated with several pro- and anti-inflammatory cytokines and analyzed for the expression of Arg-1 after 24 h of treatment. M2-polarized cells were subsequently treated with various pro- and anti-inflammatory cytokines for 24 h followed by assessment for the expression of Arg-1. (**A**) Representative contour plot of microglial cells (CD45^int^) expressing Arg-1 after treatment with various pro- and anti-inflammatory mediators. (**B**) Representative graph showing the expression of Arg-1 after treatment with select pro- and anti-inflammatory mediators. (**C**) Graphical representation of Arg-1 expression in IL-4 treated (M2-polarized) microglia after 24 h of treatment with various pro- and anti-inflammatory cytokines. UT-untreated control. Experiments were carried out three times with duplicate wells. *** *p* < 0.001.

We have previously demonstrated in vitro microglial cell polarization following IL-4 treatment using real-time RT-PCR for arginase-1 (Arg-1) mRNA [31]. In this study, we treated microglia with several cytokines and analyzed Arg-1 expression using both flow cytometry and RT-PCR at 24 h. We observed that, of the eight different treatments, only IL-4 induced Arg-1 expression indicating M2 (alternatively activated) polarization (Figure 4A,B; Supplementary Figure S2). These M2-polarized cells (treated with IL-4 for 48 h) were then treated with both pro- and anti-inflammatory cytokines for 24 h, after which they were analyzed for the expression of Arg-1. Interestingly, these M2-polarized microglial cells maintained their Arg-1 expression, and thus their alternatively activated state, even after being exposed to various pro- and anti-inflammatory mediators (Figure 4C).

### 3.4. Cytokine-Induced PD-L1 Expression on M2-Polarized Microglia

**Figure 5:**
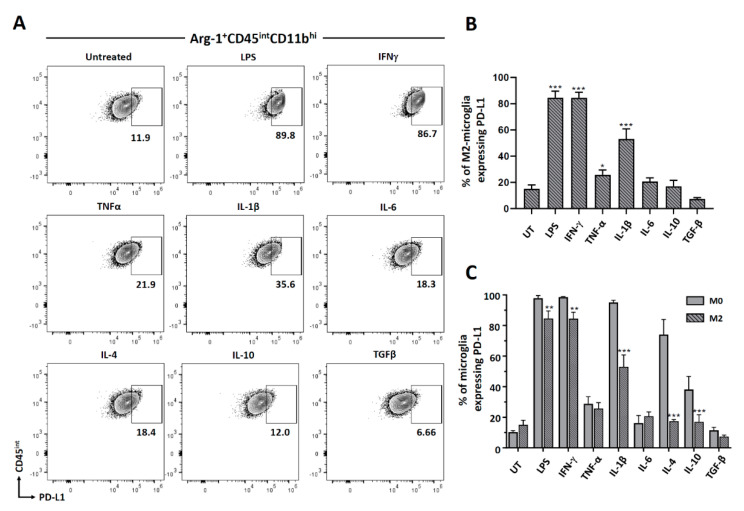
PD-L1 expression on M2-polarized microglial cells. Microglia were isolated as described previously and then treated with IL-4 to polarize them to an M2-activated state. These M2-polarized cells were subsequently treated with select pro- as well as anti-inflammatory cytokines and then analyzed for the expression of PD-L1. (**A**) Representative contour plots showing M2-polarized cells (Arg-1^+^CD45^int^ CD11b^hi^) expressing PD-L1. (**B**) Graphical representation of M2-polarized microglia expressing PD-L1 after 24 h of treatment. (**C**) Graphical representation comparing PD-L1 expression between non-polarized (M0) and M2-polarized microglia after 24 h of treatment. UT-untreated control. Experiments were carried out three times with duplicate wells. * *p* < 0.05; ** *p* < 0.01; *** *p* < 0.001.

We went on to analyze PD-L1 expression on the M2-polarized microglia. Microglia were polarized with IL-4 for 48 h and then treated with various cytokines for an additional 24 h. Cells were first gated for Arg-1 expression, as shown in Figure 4A, and then the Arg-1 positive microglial cells were analyzed for PD-L1 expression by flow cytometry. We observed that M2-polarized microglial cells significantly induced the expression of PD-L1 after treatment with pro-inflammatory mediators, with the exception of IL-6. However, we did not observe any PD-L1 induction on these cells following treatment with anti-inflammatory cytokines (Figure 5A,B). Interestingly, when we compared the expression of PD-L1 on non-polarized (M0) as well as M2-polarized microglial cells, we observed significantly less expression of PD-L1 on M2-polarized cells when compared to M0-microglia after treatment with both pro- and anti-inflammatory cytokines with the exception of TNF-α, Il-6, and TGF-β (Figure 5C).

### 3.5. Polarization of Microglial Cells to Classically Activated State (M1)

**Figure 6:**
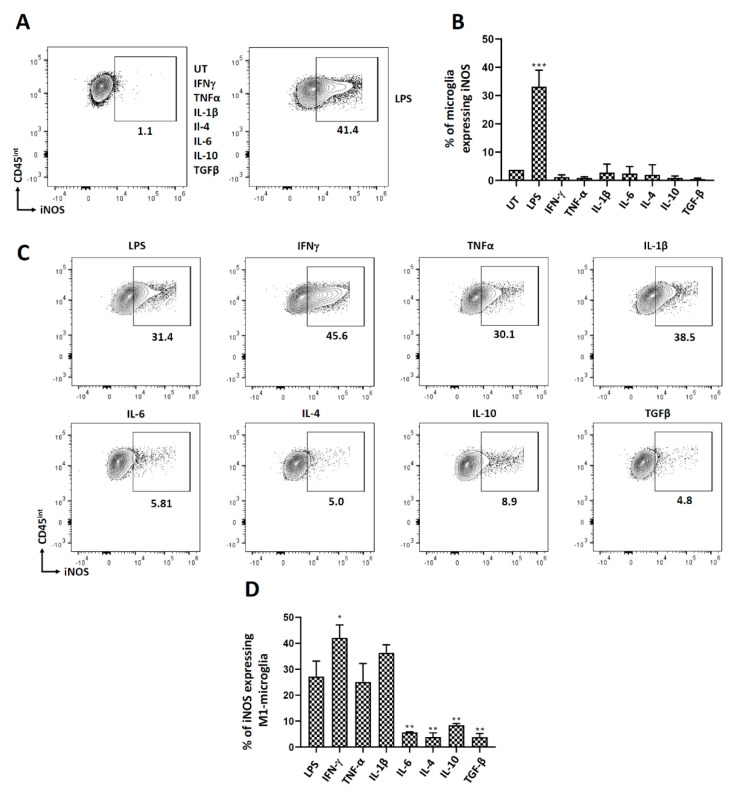
iNOS expression in microglial cells. Microglial cells were cultured from d7-10 pups as described previously, treated with select pro- and anti-inflammatory cytokines, and analyzed for iNOS expression after 24 h of treatment. Microglial cells polarized to the M1 state were also treated with various pro- and anti-inflammatory cytokines for 24 h followed by assessment of iNOS expression. (**A**) Representative contour plot of microglial cells (CD45^int^) expressing iNOS after treatment with various pro- and anti-inflammatory mediators. (**B**) Representative graph showing the expression of iNOS after treatment with different pro- and anti-inflammatory mediators. (**C**) Contour plots and (**D**) graphical representation of iNOS expression on M1-polarized microglia after 24 h of treatment with select pro- and anti-inflammatory cytokines. UT-untreated control. Experiments were carried out three times with duplicate wells. * *p* < 0.05; ** *p* < 0.01; *** *p* < 0.001.

We also wanted to investigate cytokine-induced polarization of microglia to the M1 state. For this, we analyzed iNOS expression after treatment with both pro- and anti-inflammatory cytokines using both flow cytometry and RT-PCR. Interestingly, we observed that only LPS induced iNOS expression after 24 h of treatment, indicating their M1 (classically activated) state (Figure 6A,B; Supplementary Figure S2). Further, when these cells were polarized to M1 using LPS and then treated with either pro- or anti-inflammatory cytokines, we observed that IFN-γ significantly potentiated the % of microglial cells expressing iNOS (Figure 6C,D). Interestingly, when these M1-polarized microglia were treated with either IL-6 or the anti-inflammatory cytokines (IL-4, IL-10, and TGF-β), they returned to their non-polarized state, as demonstrated by their significantly reduced expression of iNOS (Figure 6C,D).

## 4. Discussion

Resident brain cells and their microenvironment actively modulate immune responses [16,32]. Microglia display many characteristics similar to peripheral macrophages and they are considered sentinels that can orchestrate potent inflammation [33]. They respond to infection and injury by changing morphology and migrating. Their activation depends on disease context and factors such as aging, environment, or cell-to-cell interaction [33]. In this study, we aimed to elucidate microglial activation and polarization in response to select cytokines. Cytokines are small signaling polypeptides that play a pivotal role in disease [3]. They display multifaceted interactions involved in disease pathogenesis. Recent advances in cytokine biology have led to the development of new anti-viral therapies based on mimicking cytokine networks [3].

In this study, we cultured and treated primary murine microglial cells with several prototypical pro- and anti-inflammatory cytokines. First, we analyzed the expression of PD-L1 on resting non-polarized microglial cells using flow cytometry. We observed that both pro- and anti-inflammatory cytokines enhanced PD-L1 expression, however, the levels were different. These results are consistent with our previous report where IFN-γ, TNF-α, and IL-1β induced PD-L1 expression on primary cultured microglia (from d1 pups) as shown by real-time RT-PCR [28]. Others have also positively correlated IFN-γ level with PD-L1 expression in murine glioma cells, bone marrow-derived macrophages, and primary cultured microglia [34]. We did not observe a significant percentage of microglia expressing PD-L1 after treatment with IL-6 or TGF-β. Reports suggest that IL-6 signaling is important for PD-L1 expression after viral infection, but this may not be the case in vitro, as shown in our study [35]. Similarly, TGF-β induces PD-L1 expression in murine fibroblasts, but this has not been shown in microglial cells [36]. It has been previously shown that IL-4 upregulates mRNA for PD-L1 in human monocytes [37]. Investigations into the role of IL-10 have been mainly restricted to PD-1 expression on tumor-associated dendritic cells (DCs) and bone marrow-derived DC [38]. In this study, we show that IL-4, as well as IL-10, induces the expression of PD-L1 on microglia (Figure 1).

We have previously demonstrated the expression of MHC-II and MHC-I on primary microglial cells isolated from d1 pups following IFN-γ treatment [28]. In this study, we expanded our investigation to assess the effects of additional cytokines (as well as IFN-γ) using primary microglia isolated from d7-10 old pups (using the Miltenyi adult dissociation kit and CD11b selection). We examined IFN-γ, TNF-α, IL-1β, IL-6, IL-4, IL-10, and TGF-β; and established that IFN-γ most robustly induced the expression of both MHC-II and MHC-I (Figure 2 and Figure 3). However, MHC-II was expressed within 24 h of treatment, while MHC-I expression took 72 h. Besides IFN-γ, TNF-α and IL-1β treatments also upregulated MHC-I on microglial cells after 72 h, but the expression observed was significantly less than that induced by IFN-γ. IFN-γ-induced expression of MHC-II has also been previously demonstrated on mouse macrophage cell lines, bone marrow-derived macrophages, and astrocytes [39,40,41]. It has also been previously shown that IFN-γ upregulates MHC class I on primary murine neurons after 72 h of treatment [42].

Investigators will encounter a body of published work regarding the terminology of macrophage/microglial polarization, mainly into the M1 and M2 state. This phenotypic characterization was adopted to simplify the interpretation of data [43]. To investigate the role of cytokines in polarizing microglial cells to the M1 or M2 state, we analyzed cells for expression of iNOS and Arg-1, respectively. Similar to our previous report, IL-4 was found to drive microglial polarization to M2 [31]. However, in this study, we have more comprehensively analyzed a panel of pro- and anti-inflammatory cytokines. Besides IL-4, no other cytokine was found to drive polarization to the M2 state (Figure 4). Interestingly, when these M2-polarized microglial cells were further exposed to pro- and anti-inflammatory cytokines, they retained their M2 state, as indicated by Arg-1 expression. Hence, these M2-polarized cells were found to be quite refractory to depolarization. These M2-polarized microglial cells do significantly express PD-L1 after treatment with pro-inflammatory stimulants (LPS, IFN-γ, TNF-α, and Il-1β), but not after being exposed to anti-inflammatory cytokines. On comparing PD-L1 expression between M0- and M2-polarized microglial cells, we observed a significant decrease in the percentage of M2-polarized cells expressing PD-L1 after treatment with both pro- (LPS, IFN-γ and IL-1β) and anti- (IL-4 and IL-10) inflammatory stimulants as compared to M0-microglial cells (Figure 5). It has been shown in the case of macrophages that PD-L1 is expressed on both M1 and M2 cells, and there is an increased expression when the macrophages are polarized from M2 to M1 [44].

**Figure 7:**
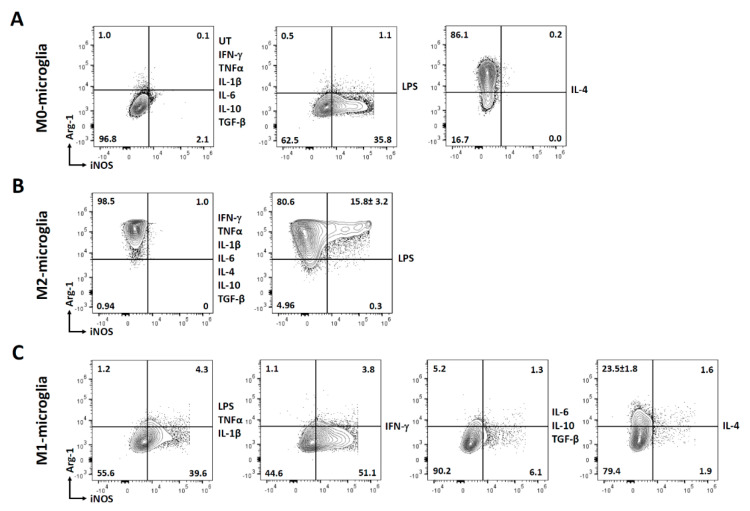
Arg-1 and iNOS expression in polarized and non-polarized microglial cells. Cells were divided into three groups: non-polarized (M0), M2-polarized (treated with IL-4), and M1-polarized (treated with LPS). These cells were then treated with various pro- and anti-inflammatory cytokines for 24 h, followed by staining for both iNOS and Arg-1. (**A**) Representative contour plots of M0-microglial cells expressing Arg-1 and iNOS after treatment with various pro- and anti-inflammatory mediators. (**B**) Representative contour plots of M2-microglia expressing Arg-1 and iNOS after treatment with various pro- and anti-inflammatory mediators. (**C**) Representative contour plots of M1-microglial cells expressing Arg-1 and iNOS after treatment with various pro- and anti-inflammatory mediators. UT-untreated control. Experiments were carried out three times with duplicate wells.

We went on to analyze the polarization of microglia to an M1 state. Surprisingly, only LPS treatment was found to activate microglial cells to M1. This is contradictory to what we have observed previously where IFN-γ polarized microglia to the M1 state [31]. This could be attributed to the fact that microglial cells were isolated in a different way and from different aged mice in these two studies. When these M1-polarized microglial cells were exposed to pro- inflammatory cytokines, they maintained their M1 state. Moreover, IFN-γ significantly potentiated the number of microglial cells expressing iNOS, thereby the number of M1 polarized cells. Most interestingly, when these M1-polarized microglial cells were exposed to anti-inflammatory cytokines, they returned to the M0 state. Hence, the M1 state of microglial cells is very sensitive to depolarization and environmental stimuli (Figure 6 and Figure 7).

## 5. Conclusions

In summary, we demonstrated that LPS treatment polarized microglia to an M1 state, as shown by increased iNOS expression (and no Arg-1 expression), while IL-4 polarized the cells to an M2 state as shown by increased Arg-1 (and no iNOS expression) (Figure 7A). When the M2-polarized microglial cells are exposed to various pro- and anti-inflammatory cytokines, these cells stay M2-polarized (as indicated by positive Arg-1 expression and no iNOS expression). They appear to be quite resistant to depolarization. However, approximately 15% of M2-polarized microglial cells do exhibit an intermediate state of activation after treatment with LPS, as shown by both positive Arg-1 and iNOS staining (Figure 7B). On the other hand, microglial cells (pretreated with LPS) remain M1-polarized after additional treatment with IFN-γ, TNF-α, and IL-1β (as shown by positive iNOS expression). IFN-γ significantly increased the percentage of microglial cells expressing iNOS and thereby the % of M1-polarized microglial cells (Figure 7C). Interestingly, when these M1-polarized microglial cells were treated with either IL-6 or the anti-inflammatory cytokines (IL-10 and TGF-β), they returned to their non-polarized state (negative iNOS and Arg-1 expression). Most importantly, when the M1-polarized microglial cells were treated with IL-4, a percentage of these cells de-polarized towards an M2 state (indicated by positive Arg-1 expression) (Figure 7C). Although, classification into M1 and M2 polarization states has been helpful for conceptualizing microglial activities in vitro, it is increasingly acknowledged that the M1/M2 paradigm is oversimplified, artificial, and inadequate to describe the many states of microglial cell activation in vivo [43]. There is always a question about the relevance of classifying cell polarization as M1 and M2, as there are likely multiple phenotypes of microglia [45].

Limitation of the study: it is a comprehensive in vitro study investigating the modulation of microglial cells and their polarization in response to both pro- and anti-inflammatory cytokines. Although we examined the effects of the most common select, prototypical cytokines, we were limited in the number of mediators we could study. Responses could also vary because of treatment kinetics. This study utilized microglia isolated from 7-10 d-old pups. It would be interesting to compare these findings with those obtained using cells from older mice; since aged microglia may be phenotypically and functionally different from neonatal cells [46].

## Data Availability

Data is contained within the article or supplementary material.

## Supplementary Materials

The following are available online at https://www.mdpi.com/article/10.3390/brainsci11111482/s1, Figure S1: MHC-I expression on non-polarized (M0) microglial cells, Figure S2: Arg-1 and iNOS expression in microglial cells by real-time RT-PCR.
