# Supplementary material for: Differential Cytokine-Induced Responses of Polarized Microglia
Source: Brain Sci. 2021 Nov 10;11(11):1482. doi: 10.3390/brainsci11111482 (PMC8615503; doi:10.3390/brainsci11111482)
Supplement: Supplementary file 1 [file brainsci-11-01482-s001.zip › brainsci-1430491-supplementary.pdf]

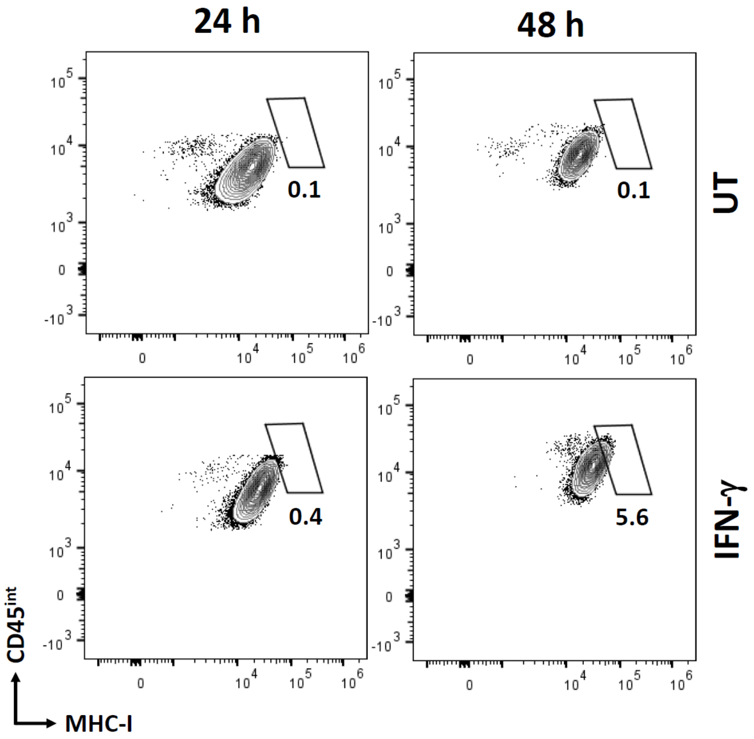

Figure S1. MHC-I expression on non-polarized (M0) microglial cells. Microglial cells were treated with IFN-γ for 24 h and 48 h after which they were trypsinized and then stained for MHC-I. Representative contour plots showing microglial cells (CD45int) expressing MHC-I. UT-untreated control. Experiments were carried out twice with duplicate wells.

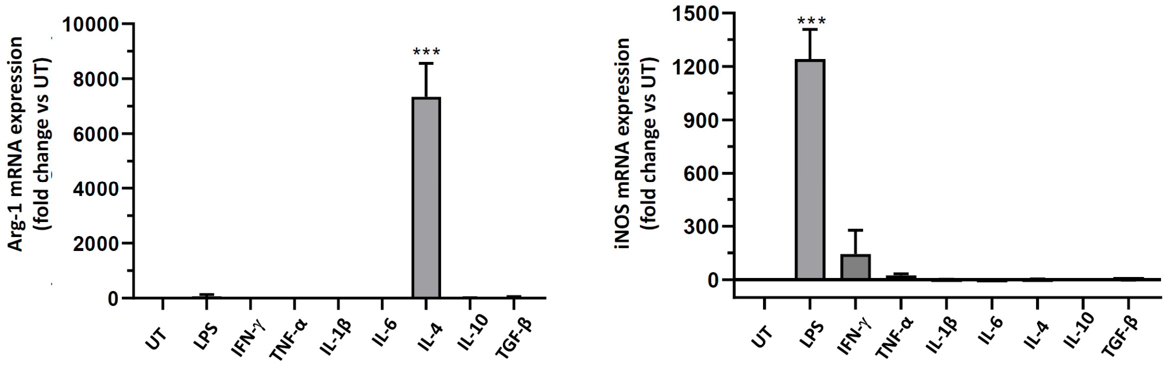

Figure S2. Arg-1 and iNOS expression in microglial cells by real time RT-PCR. Microglial cells were cultured and treated with select cytokines as described. cDNA synthesized from 0.5 µg -1 µg RNA was amplified and fold change of mRNA expression of Arg-1 and iNOS relative to unstimulated control was quantified using the $2^{-\Delta\Delta Ct}$ method and normalized to the housekeeping gene HPRT. UT-untreated control. Data shown are mean ±SE of 3 experiments. ***p<0.001.
